# Ultrasound diagnosis of non-mass MRI-detected lesions

**DOI:** 10.1007/s10396-023-01306-x

**Published:** 2023-04-29

**Authors:** Ayumi Izumori, Yumi Kokubu

**Affiliations:** 1 Department of Breast Surgery, Takamatsu Heiwa Hospital, Takamatsu, Japan; 2 Department of Ultrasound/IVR Diagnostic Imaging Center, The Cancer Institute Hospital of Japanese Foundation for Cancer Research, Tokyo, Japan

**Keywords:** MRI second-look ultrasonography, MRI-targeted US, Non-mass enhancement (NME), Anatomical landmark, LAFS/PAFS

## Abstract

Magnetic resonance imaging (MRI)-detected lesions are often category 2 or 3 lesions on initial ultrasound examination. In addition, in the case of new non-mass lesions detected on MRI, one would expect to find lesions with ductal dilatation with minimal secretory accumulation, single short lesions with ductal dilatation, cyst-like lesions less than 5 mm in size, mammary gland-like lesions less than 8 mm in size, and very indistinct lesions. Detection is expected to be even more difficult. Currently, there are no clear uniform criteria for the indication of second-look ultrasonography (US) for MRI-detected lesions, so it is not possible to make a general comparison, but recent studies have indicated that the ratio of mass to non-mass MRI-detected lesions is 7:3. And it has been pointed out that the percentage of malignancy is about 30% for each. Before about 2012, the US detection rate was about 70%, and MRI-guided biopsies of undetected lesions showed a small percentage of malignant lesions. Therefore, some observers believe that lesions not detected on US should be followed up, while others believe that MRI-guided biopsy should be performed. Recently, however, the use of surrounding anatomical structures as landmarks for second-look US has increased the detection rate to as high as 87–99%, and the percentage of malignancy remains the same. In addition, recent surveillance of high-risk breast cancer requires careful management of MRI-detected lesions. In this review, we will discuss the literature on MRI-detected lesions and describe ultrasound techniques to accurately detect small lesions and reliably reveal pale lesions based on their structural differences from their surroundings.

## Introduction

Breast magnetic resonance imaging (MRI) is used as a routine diagnostic tool for preoperative diagnosis of cancer extent, preoperative evaluation of the contralateral breast, chemotherapy efficacy determination, close examination of abnormal nipple blood secretion, and when other breast imaging studies yield suspicious results. Recently, insurance coverage for the treatment of patients with hereditary breast and ovarian syndrome (HBOC) was extended in Japan (in 2020), and breast MRI surveillance is now being performed [1]. MRI-guided biopsy is the most accurate means of diagnosing MRI-detected lesions, but due to the challenges of time, cost, and facility requirements, many do not have the opportunity to undergo such examination. In addition, more convenient real-time virtual sonography (RVS) devices increase the diagnostic rate [2], but they require supine MRI imaging or computed tomography (CT) images and are not yet widely used. Most newly detected lesions on MRI are benign, with malignant lesions accounting for 10–30% [3–5]. In addition, those ultrasound (US) images show category 2 lesions or show benign variant images that make it difficult to point out their presence as lesions [6]. Previous studies have shown that US findings of MRI-detected lesions cannot be used to differentiate between benign and malignant lesions. Therefore, the US diagnosis of MRI-detected lesions can be said to include accurate identification of MRI-detected lesions and reliable US-guided intervention.

This review describes lesions that are not noted as abnormal findings on first-look US but are newly detected on MRI (MRI-detected lesions) and the ultrasound techniques required for second-look US.

### About terminology

MRI is a test with high sensitivity and low specificity [5]. Therefore, breast cancers that could not be detected on mammography (MG) or US could now be detected, and there was a phase in early 2007 when they were called "occult cancer" [7]. Since then, research on lesions newly detected on MRI has progressed, and the term "MRI-detected lesion" has recently become established for these lesions. In Europe and the United States, MG is used for screening, and US is performed by targeting the site of MG findings and clinical symptoms, which is called "target ultrasound" [8, 9]. In addition, US examinations in which lesions detected on US are newly evaluated using contrast-enhanced ultrasound [10] or automated breast ultrasonography (ABUS) are also referred to as "target ultrasound" [11]. For this reason, US examinations performed on MRI-detected lesions are also called "MRI-targeted US" [12]. On the other hand, US is also used for screening and follow-up in Asia. US performed on lesions newly detected on MRI that were not selected for close scrutiny on first-look US is referred to as "MRI second-look US”. The terms "MRI-targeted US" and "MRI second-look US" are not unified.

### Criteria for MRI-detected lesions

There are no uniform criteria for detection of lesions on MRI or for the indication of second-look US, even among radiologists [13]. Many reports have evaluated morphology according to BI-RADS. Some reports have included empirical evaluation by radiologists. Some have looked at "early rise and early washout" in kinetic curve assessment [9, 14, 15], while others state that "kinetic curve assessment is not a question" [16]. Some listed shapes as “spiculated or indistinct borders, heterogeneous or rim enhancement, and interval change since prior breast MRI examinations” [17] or obscure lesions including “masses with irregular shape, irregular and spiculated margin, and marked enhancement, and non-mass-like enhancements with ductal and clumped enhancement patterns” [4], while others targeted C4 and C5 according to the BI-RADS MRI diagnostic procedure [18–20]. Some reports include all contrast-enhancing effects regardless of shape [21], while others include benign lesions [22].

### Percentage of MRI-detected lesions

Thus, the percentage of MRI-detected lesions varies from report to report because uniform criteria have not been established (Table 1) [4, 9, 14–19, 23–28]. There is a report of 69 new lesions (46%) detected in 149 preoperative MRI examinations of breast cancer patients [9], and another report of 149 lesions (7.6%) detected in 1970 preoperative and postoperative MRI examinations of breast cancer patients [4]. Since 2008, there have been some reports separating mass and non-mass enhancement (NME), with new NME noted on MRI in 6–34% of ipsilateral and 3–5% of contralateral lesions [29–31]. In all reports, NME is detected less often than masses, ranging from 8.6–31 to 61–91.4% [3, 4, 14, 17, 25, 28, 31].

**Table 1:** Comparison of detection rates and second-look US landmarks, detected lesion size, and US models

	Detection rate (%)	US landmark	MRI-detected lesion size (mm) (range)	MRI-detected lesion NME size (mm) (range)	US model	US probe frequency
Linda [17]	23	–	9 (3–50)	29	Siemens Acusion,	7–10 MHz
					Philips HDI 5000	
Sim [23]	66.7	–	11 (3–26)	–	Toshiba SSA 380	10 MHz
Shin [9]	71.1	–	11 (4–50)	–	GE Logiq 700,	7–10 MHz
					Philips HDI 5000	
Wiratkapun [24]	47.2	–	7 (3–44)	–	Siemens sonoline antares	7–11 MHz
Meissnizer [25]	55.9	–	12 (2–70)	22 (6–70)	Siemens Acuson sequoia 512	8–15 MHz
Demartini [14]	45.5	–	16 (3–85)	–	GE LOGIQ7	12 MHz
Destounis [26]	70.2	Clock position, depth from the skin, findings, assessment of surrounding tissues	9.5 (4–85)	–	Philips iU22,	12.5 MHz
					Siemens sonoline antares	13.5 MHz
Abe [15]	56.9	–	–	22.8	Philips ATL HDI 5000	5–12 MHz
Carbognin [18]	71	Distance from the nipple, depth from the skin, findings	–	–	Esaote Technos MPX	7–11 MHz
Candelaria [19]	67.2	Clock position, distance from the nipple, depth from the skin	10 (3–50)	–	Siemens sonoline antares	5–13 MHz
Laguna [16]	61.8	–	6.8 (3–22)	–	Philips HD11XE	7–12 MHz
Kim [21]	40	Position, findings	–	32.6 ± 7.4	Siemens Acuson sequoia 512	15 MHz
Aracava [27]	67.6	Distance from the nipple, depth from the skin, findings	9 (3–92)	–	Philips HDI 5000,	10–12 MHz
					Toshiba,	
					GE Logiq 700	
Hong [4]	86.8	Findings, subcutaneous fat, glandular tissue, subglandular fat	12 (5–60)	24.4 (7–60)	Philips IU22	5–12 MHz
Izumori [28]	99	Clock position, benign lesion, mein lesion, findings, glandulae pattern, fat, mammary fascia, vascular routes	9 (3–82)	17.8 (3–82)	Canon Aplio 500	14 MHz

### US detection rate of MRI-detected lesions

The US detection rate of MRI-detected lesions varied from 23 to 71% in reports from 2003 to 2012 [9, 14–19, 21, 23–26]. A 2014 meta-analysis by Spick et al. [13] reported that identification rates were affected by malignant > benign and mass > NME, and were not related to lesion size. The detection rate of NME is 12–15%. This may be because "malignant lesions have lower echo level images" and "masses are more easily recognized as lesions”. It is assumed that lesions whose echo brightness levels do not differ significantly from their surroundings or lesions with indistinct borders cannot be detected on US.

However, the detection rate was as high as 86.6% for masses and 87.5% for NMEs in a 2015 report by Hong et al. [4], and 99% (98% for masses, 100% for NMEs, 100% for foci) in a 2019 report by the present authors [28]. The lesion sizes were 8.0 mm (5–33 mm) for masses, 17.8 mm (3–82 mm) for NMEs, and 4.2 mm (3–5 mm) for foci [28], which were smaller than the average size of 8.5–10.1 mm for masses and 22–32.6 mm for NMEs [4, 9, 14, 15, 19] in the literature to date. Despite the fact that the study targeted smaller lesions than the average size of 8.5–10.1 mm for masses and 22–32.6 mm for NMEs [4, 9, 14, 15, 19], high detection rates were obtained. One reason for this is an understanding of anatomical landmarks and breast deformities (see below).

### Malignancy rate and histology of MRI-detected lesions

Comparing US-detected versus undetected lesions by the malignancy rate of MRI-detected lesions in the 2003–2012 report, there was more cancer (43% vs. 14%) and more invasive cancer (78% vs. 50%) [17] among the detected lesions. In addition, reports of MRI-guided histologic examination of lesions not detected on US have shown a malignancy rate of 28–28.6% [14, 32] for all MRI-detected lesions. This malignancy rate is also consistent with the malignancy rate of 29.8% reported by the present authors in [28] above. The malignancy rates by MRI morphology reported by the authors are 17.2% for foci, 40.7% for masses, (foci + mass 27.8%), and 34.8% for NMEs. The percentages of each invasive cancer were 25.0%, 38.7%, and 13.0% (p < 0.05), respectively, and NME had more ductal carcinoma in situ (DCIS).

The malignancy rate of MRI-guided biopsy of lesions not detected on US is 9–21%, with some reports suggesting that lesions not detected on US should be considered for MRI-guided biopsy [3, 25, 27]. The malignancy rate of MRI-guided biopsy in Japan since 2010 has been reported to be 36–38% [12, 33, 34]. It is possible that the detection rate of MG is lower in Japan because of the high-density mammary glands of the population, and that even in the case of US, lesions may be masked in the structural pattern in mammary glands with abundant surrounding stromal volume [35, 36]. These relationships need to be studied in the Japanese population. Also, according to a study by Chikarmane et al. [37], the malignancy rate in a high-risk population of MRI BI-RADS category 3 lesions with BRCA pathological variants or a history of breast cancer was 3.8% (11/288), and 0% (0/147) in a population without a family history or history of breast cancer. The malignancy rate is likely to vary depending on the background of high-risk breast cancer.

### NME characteristics of MRI-detected lesions

The histological type of NMEs is listed as focal adenosis, fibro-cystic, radial scar, complex sclerosing lesion, inflammatory changes, intraductal papilloma, flat epithelial atypia, lobular carcinoma, diffuse invasive breast cancer, invasive ductal carcinoma, and DCIS [38–41].

NME is noted as the primary lesion in about 20% of cases, of which 20.8% are malignant and 84% are noninvasive ductal carcinoma of the breast [41, 42]. On the other hand, DCIS is often noted as a lesion with calcification on MG, and DCIS with a high histological grade, such as those with necrosis, are characterized by characteristic non-mass images on MRI findings, which are highly suspicious of malignancy [43–49]. Such DCIS are associated with hypoechogenicity, reflecting stromal thickening, and are easily noted on first-look US [50]. DCIS with no findings on MG and category 2 on first-look US are often small masses similar to cysts about 5 mm in size [51]. They are noted as foci on MRI.

Therefore, in the case of NMEs for MRI-detected lesions, one would expect to find lesions with ductal dilatation with minimal secretory accumulation, single short lesions with ductal dilatation, cyst-like lesions less than 5 mm in size, mammary gland-like lesions less than 8 mm in size, and very indistinct lesions.

## Landmarks

Breast MRI must be performed in the supine position the first time. Failing to do so will result in reduced spatial resolution and difficulty diagnosing the cancer extent [52, 53]. Therefore, when performing US examinations, the breast is deformed in the back-lying position, making it difficult to correlate with small lesions. In an attempt to match an MRI-detected lesion to its US counterpart, spatial and regional displacement and distance from the nipple are considered, with the deformity being a circumferential movement in a solitary circle around the nipple [54], and methods of measuring lesion location using anatomy such as the sternum or tracheal bifurcation [55] are being attempted. However, even with these methods, small lesions and DCIS are considered undetectable.

**Fig. 1:**
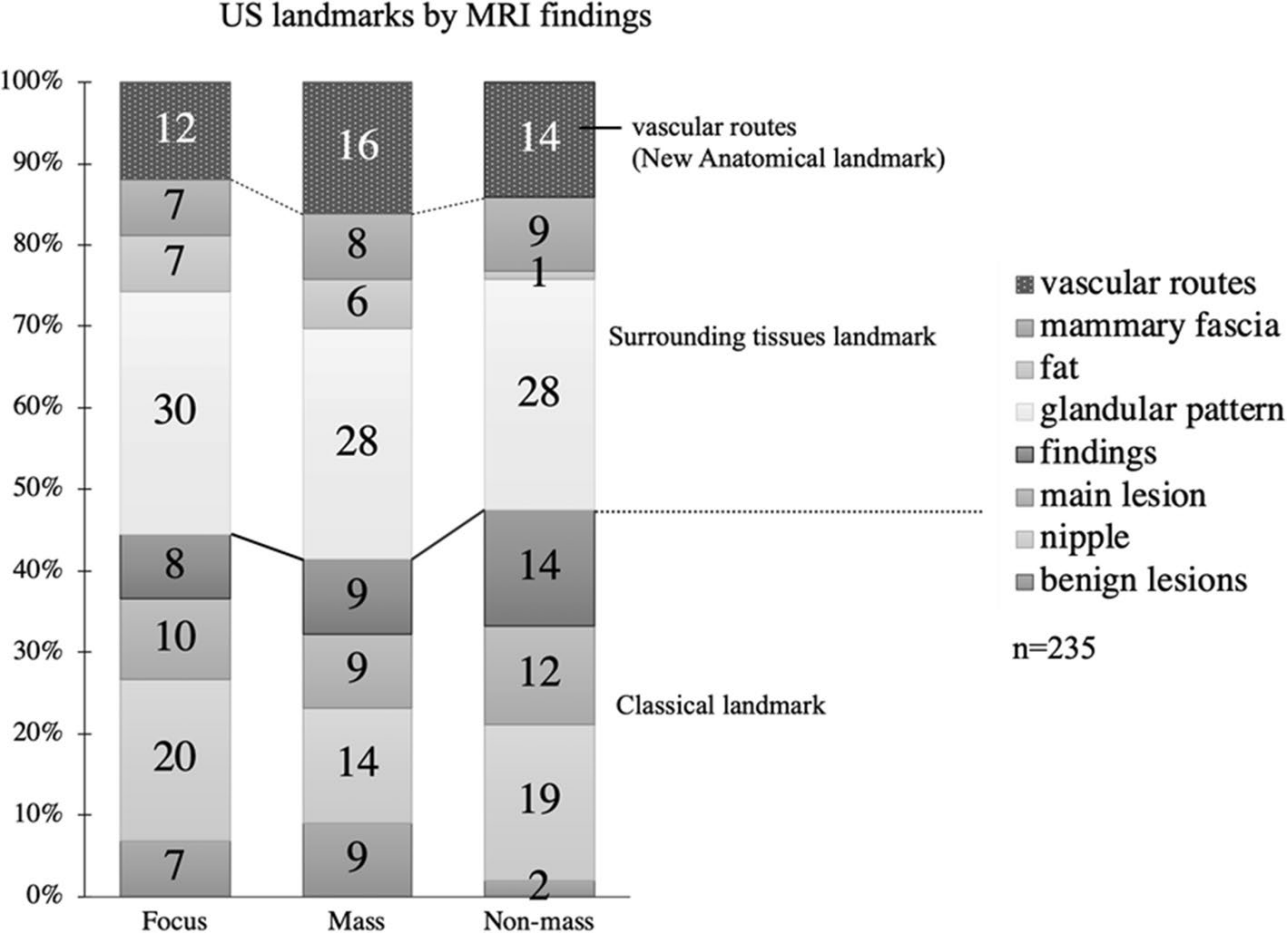
Adapted from Reference [28]. The main indicators that led to the identification of targets on US. The main US indicators showed the same trend regardless of the MRI findings. A glandular pattern was the most common landmark, accounting for 28–30% of the lesions identified. New anatomical landmarks, i.e., vascular routes, led to 12–16% of the lesions identified

Table 1 summarizes detection rates and second-look US landmarks. Landmarks used in reports with detection rates of 23–71% are (1) shape, clock indication, distance between papillary tumors, benign lesions such as cysts, and depth. The present authors reported this classical landmark [28]. As mentioned above, the lesions identified based on these are malignant > benign and mass > NME, which may also be due to the fact that they were identified primarily by relying on shape. In Hong's report [4] with a detection rate of 86.8%, (2) mammary gland distribution, shape, fat, and Cooper's ligament were added to the landmarks, which the authors reported as surrounding tissue landmarks. In a report by the present authors [28] with a detection rate of 99%, (3) vascular routes from the axilla to the outer breast, perforating branches of the internal thoracic artery, and vessels showing a characteristic run were used, and this was reported as (3) vascular routes (new anatomical landmark). When the landmarks used are examined for each MRI geometry, the detection rate is about 45% for landmark (1), which is the same for all geometries, and about 85% by adding landmark (2), and the detection rate trend is consistent with previous reports. By adding landmark (3), a detection rate of 99% is achieved (Fig. 1).

### Anatomical understanding of breast deformity

To effectively use the (2) surrounding tissues landmark and (3) vascular routes (new anatomical landmark), it is necessary to understand the anatomy of the "pattern of fat and mammary gland distribution in the breast" and " deformation and displacement” [28].

**Fig. 2:**
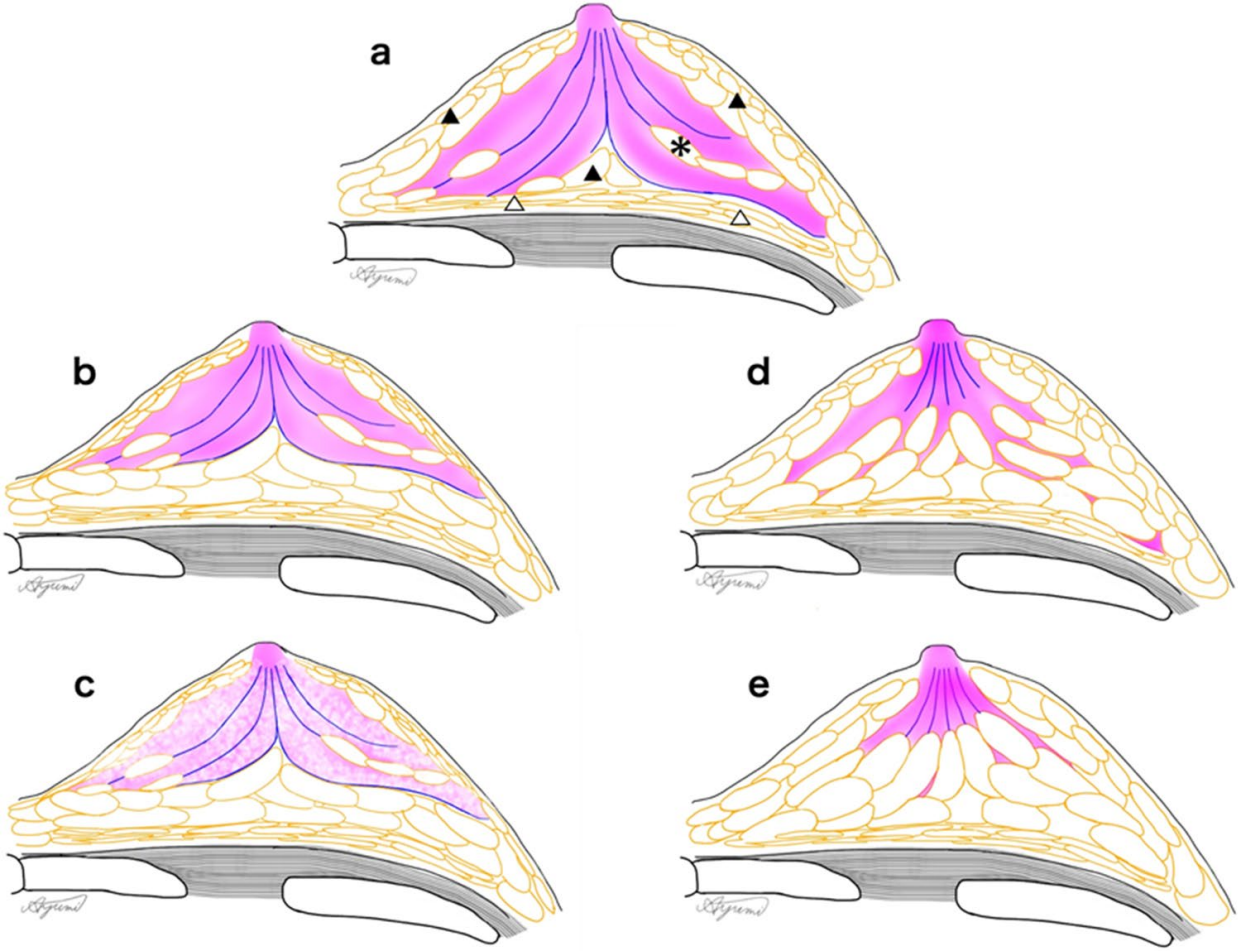
Adapted from Reference [28]. Image for understanding fat and mammary gland distribution in breast image. **a** The fibroglandular zone is distributed to the periphery. The mammary lateral and posterior border lines are clear. Some fatty lobules (asterisk) are partially visible between the glandular lobes. PAFS: protective adipofascial system (filled triangle), LAFS: lubricant adipofascial system (open triangle). **b** There is abundant mammary posterior fat. The mammary lateral and posterior border lines are clear. **c** Fat is mixed in the fibroglandular zone. The mammary lateral and posterior border lines are clear. **d** The fibroglandular tissues are distributed between multiple fat lobules, and the lateral and posterior fat boundaries are ambiguous. **e** The fibroglandular tissues are mainly distributed near the nipple, and the fibroglandular tissues between the fat lobules are barely visible. Sometimes, mammary gland distribution patterns shown in (**a**–**e**) are mixed in one breast. Use the technique in Fig. 4 to detect the fatty breast lateral borderline (d and e dotted circles) on US images

First, "pattern of fat and mammary gland distribution in the breast " are described. The breast is glandular tissue distributed within the subcutaneous fascial layer and anatomically developed in the gaps between fat lobes called the protective adipofascial system (PAFS) [28, 56, 57] (Fig. 2). If distributed from the nipple to one continuous fat lobule gap, the mammary gland will be distributed in a single block pattern; if distributed in multiple gaps, the pattern will be mixed with fat. Although the distribution of mammary glands, the adiposity of the breast, and the adiposity of the mammary glands may appear complex at first glance, every breast can be understood as a combination of these fat lobes, mammary gland distribution, and adiposity. Because the size and shape of the fat lobes are not uniform but vary slightly, and the volume of the mammary gland lobes also varies, the fat shape and mammary gland distribution often take on a characteristic shape. Because the fat lobes and mammary glands of the PAFS have only negligible mobility, the features are preserved even when the breast is deformed and can serve as one-to-one landmarks that can accommodate deformation. When reading the morphology of the fat lobule and mammary gland distribution as landmarks, knowledge of the anatomy in Fig. 2 will make it easier to understand the fat lobule and mammary gland distribution.

**Fig. 3:**
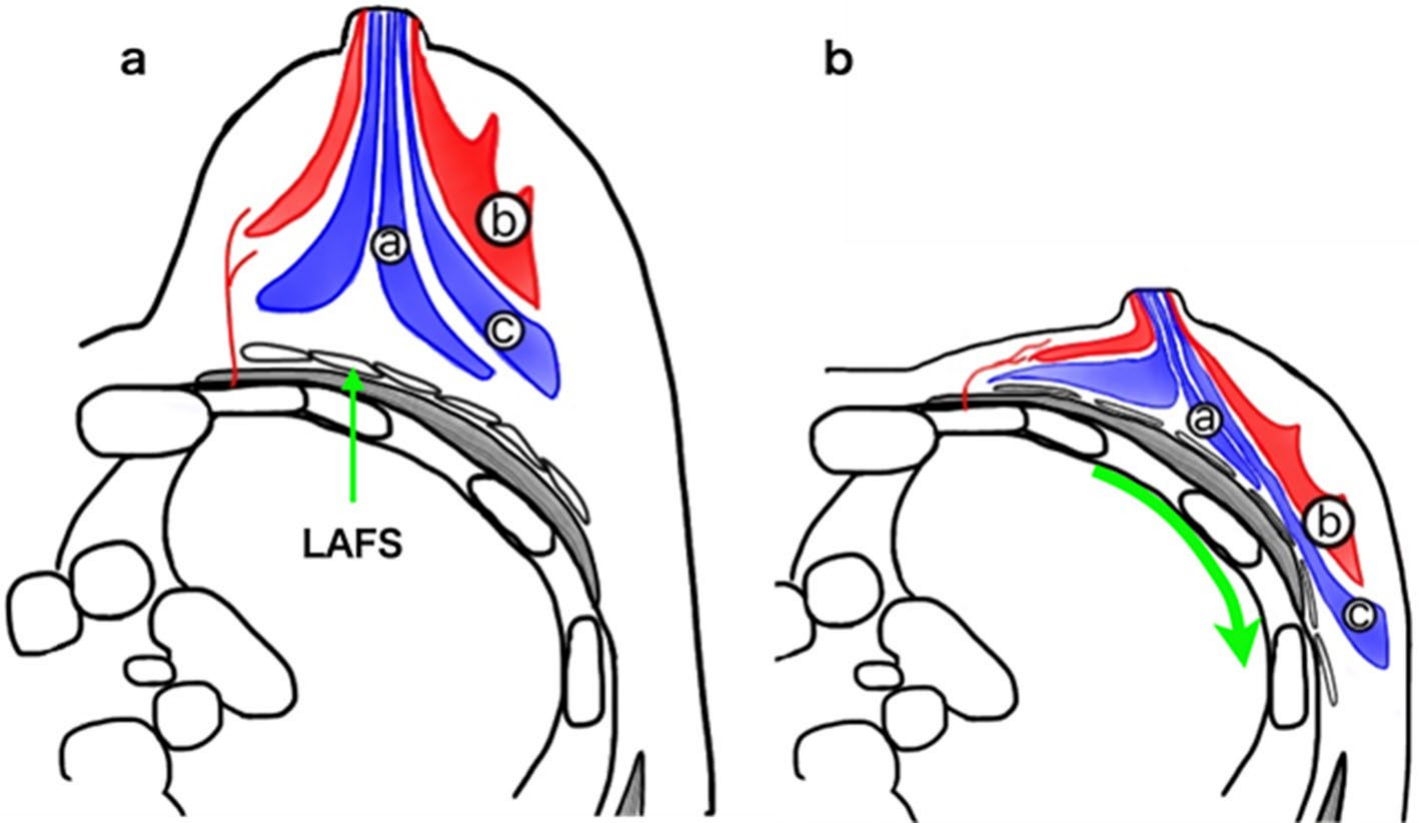
**a** On MRI, lesion a is on the border of the glandular lobe just below the nipple. Lesions b and c are aligned anteriorly and posteriorly. LAFS is present on the pectoralis major side. **b** On the US image, the pectoralis major side is displaced laterally by the LAFS for a longer distance than the cutaneous side. Lesion a is displaced lateral to the nipple. Lesion c moves a longer distance laterally than lesion b

Next, " deformation and displacement " are described. As mentioned earlier, the mammary glands are distributed in the PAFS and are fixed to the skin in the anterior position. Posterior to the mammary gland is the lubricant adipofascial system (LAFS) [28, 56, 57]. Because of the movability of the LAFS, the posterior mammary gland moves outward along the thoracic cage when the patient is in the dorsal recumbent position. And there is also a slight fibrous connective tissue between the gland lobes [35] and a little mobility [28], so that the posterior mammary gland travels a longer distance than the anterior mammary gland (Fig. 3). Consider that the degree of this deformation and displacement, i.e., the mobility of the LAFS and the degree of deformation of the fat lobes, varies from individual breast to breast and does not necessarily correspond to the size of the breast or the breast composition of fat and mammary glands [28].

**Fig. 4:**
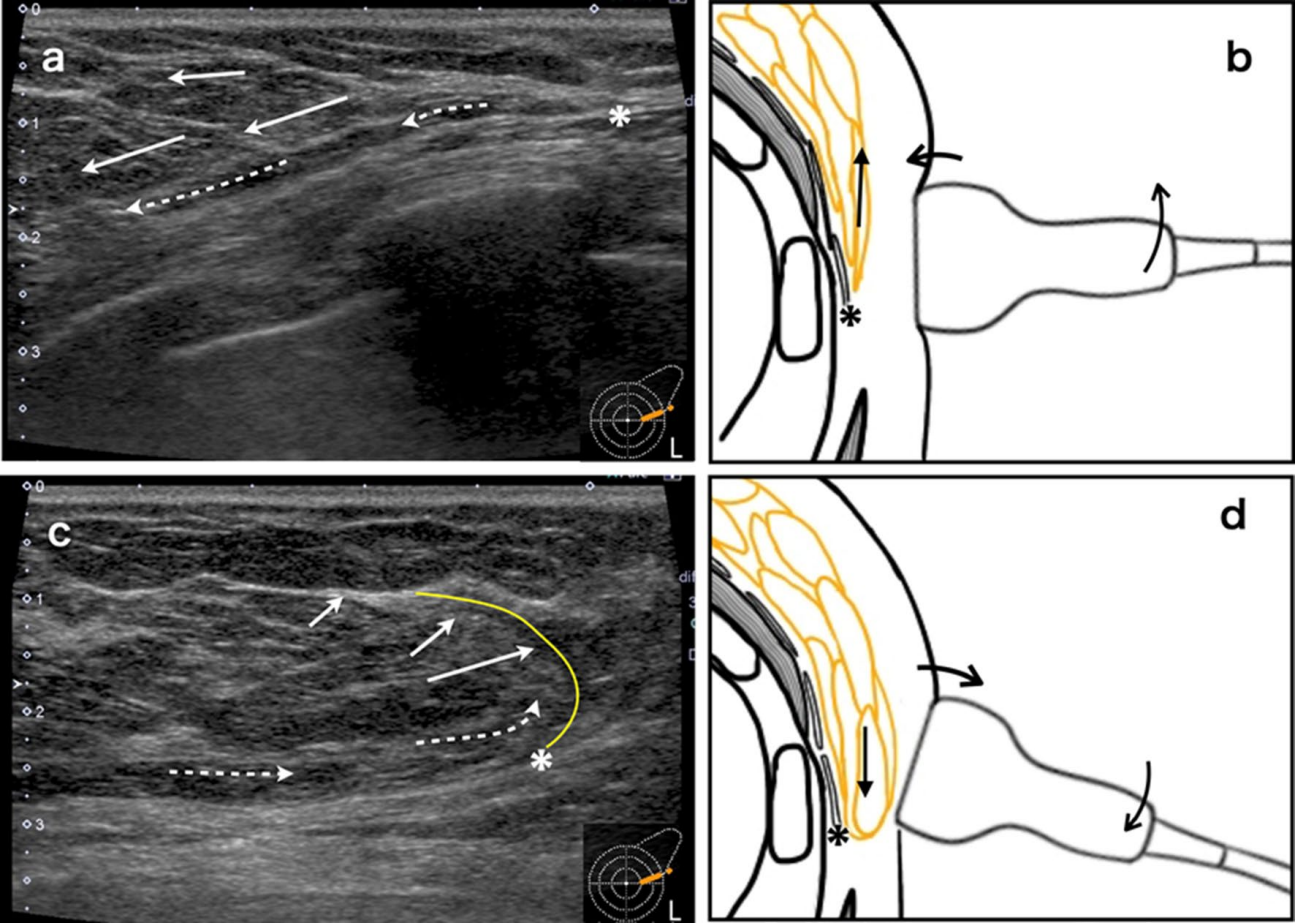
Adapted from Reference [28]. Technique for finding the lateral border line of fatty breasts. **a**, **b** With the probe pressed, the lateral border line is obscured because the dorsal fat and breast fat look similar in shape. **c**, **d** When the inner pressure of the probe is released, the adipose layer of the retro-mammary space, LAFS (dotted arrow), slides, and only the fat in the breast (arrow) is displaced outward. Then, a curve (yellow line) from the outer boundary of LAFS (asterisk) appears. Repeat (b) and (d) several times

When NME is seen in the peripheral mammary gland, the outer border of the mammary region becomes an anatomic landmark. However, in areas with more fat and fewer mammary glands (Fig. 2d, e), the outer border of the mammary region is obscured. This outer boundary is clearly visible when the mobility of the LAFS is used (Fig. 4) [28]. When the probe is compressed, the lateral border is not visible because of the similar shape of the dorsal and thoracic fat (Fig. 4a, b). When the probe pressure is released, the fat layer of the posterior breast cavity (dotted arrow) slides and only the breast fat (arrows) moves outward (Fig. 4c, d). Then, a curve (yellow line) can be seen from the outer boundary of the LAFS (asterisk). Repeating scans (b) and (d) several times will clarify the outer border of the fatty mammary gland and confirm the anatomical correlation between MRI and US peripheral mammary gland anatomy.

If the NME is a small submammary line or foci, observation of the submammary ductal run is especially important. In the back-lying position, the breast fits into a small thickness of space, and the submammary area is often occupied by the medial glandular lobe due to the mobility of the LAFS. The boundary surface between the inner and outer glandular lobes can be seen on both MRI and US, which can give an idea of the degree of inclination of the mammary gland (Fig. 3b) [28]. During US sweep observation, the interface is detected as a boundary surface that differs from the “TDLU-duct-surrounding stroma pattern”; it is occasionally confirmed as a partial sheet-like hyperechoic image [28, 35]. It should also be noted that in the position with the nipple at the apex, the glandular lobe with large volume is beyond the subnipple. It is easy to use the nipple as a landmark, but anything below the nipple on MRI is mutated either way on US, and anatomic concordance needs to be confirmed by considering the volume of the glandular lobe and the mobility of the LAFS.

### Usefulness of anatomical landmarks

Marking: New lesions are found in 20–63% of ipsilateral and 10% of contralateral surgical specimens [58–63]. The ipsilateral breast recurrence rate is 4–14% [64]. Therefore, new preoperative MRI detecting breast cancer results in a change of procedure in 11–31% of cases [61]. Accurate second-look US is also important to avoid change of technique and additional resection.

On the other hand, there are reports that there was no significant difference in the reoperation rate between the group for which MRI was used before breast cancer surgery (816 cases) and the group for which MRI was not used (807 cases) [65], and conversely, an increase in the reoperation rate (28% vs. 45%) [66]. This may be attributed to the fact that the extent of resection was determined in a situation where not all lesions could be identified at second-look US. Although the surgical position is sometimes different from that at second-look US, it is expected that reading the breast from an anatomical perspective can accommodate deformities in the surgical position and will provide superior capability in determining the extent of resection.

**Fig. 5:**
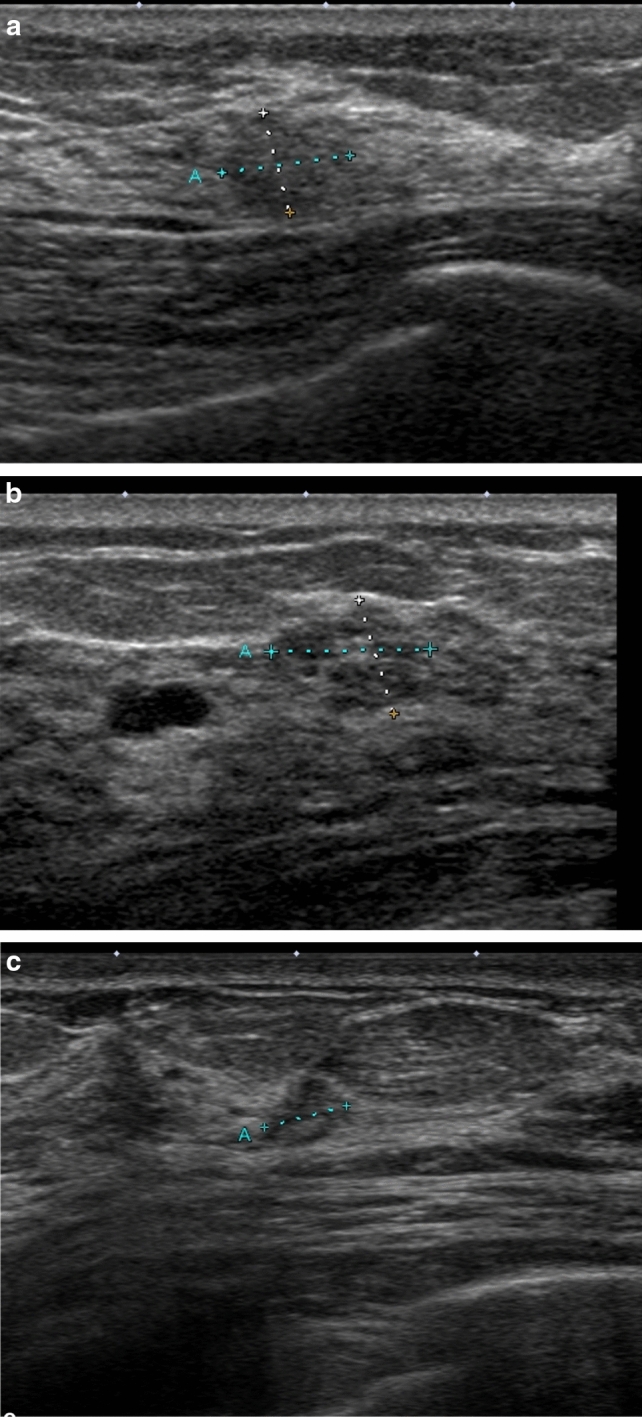
**a** Forties, MG. Extremely dense mammary glands, category 1. During 6-month follow-up for mastopathy, a new isoechoic lesion occurred at 3 o’clock. T = 6.9 × 5.5 mm. Assessed as malignant based on fine needle aspiration cytology (FNA). **b** In case a, the newly detected lesion at 12 o’clock on MRI was also isoechoic and similar to mastopathy. T = 8.8 × 6.6 mm. Assessed as malignant based on FNA. Both were determined to be invasive ductal carcinoma, NG3, lymph node metastasis 1 + . **c** Sixties, MG. Scattered mammary glands, category 1. Invasive cancer was diagnosed in the left breast. Preoperative MRI was performed, and a new lesion was detected in the right breast. A very indistinct lesion was detected, T = 4.7 × 3.5 mm. Assessed as malignant based on FNA. Invasive ductal carcinoma, NG1

Follow-up: At follow-up, it is necessary to observe the same areas as before without mistake and to notice the appearance of indistinct pale lesions. Depending on the histologic composition, such as invasive carcinoma with a predominant intraductal component, the lesion may not be at a hypoechoic level, and isoechoic lesions may be indistinguishable from the surrounding area at small sizes (Fig. 5). The present authors reported that to observe MRI-detected lesions, it is necessary to detect isoechoic lesions and small non-mass lesions, rather than detecting only hypoechoic lesions [35]. To easily detect them, we have proposed an observation method to understand the normal structure of the mammary gland and to detect deviations from the normal structure [35]. However, isoechoic lesions and small non-mass lesions are difficult to identify with only the "shape, clocked from the nipple" landmarks in a deforming breast. Using anatomic landmarks, it is mostly possible to identify the site of MRI contrast even when there is no clear hypoechoic lesion on US. If the site of the MRI-detected lesion can be accurately identified, it is possible to recognize the slight changes that appear there as "deviations from the normal structure". We believe that anatomic site identification and lesion detection will make follow-up more accurate and objective than US and reduce the number of MRI follow-ups.

### Future challenges for MRI-detected lesions

MRI-detected lesions are lesions that have not been the subject of close examination on first-look US, so the US BI-RADS category is also often 2 or 3. It is difficult to distinguish between benign and malignant based on conventional US imaging [16, 67]. Therefore, interventional diagnosis is required for all those detected.

Future issues include the following. (1) Whether the use of US equipment, such as high-frequency probes (18–24 MHz), will enable morphological distinction between invasive and noninvasive cancer, and whether low blood flow display and contrast-enhanced US will increase the malignant diagnosis rate compared to MRI. (2) Distinguishing between background parenchymal enhancement and NME is difficult even for radiologists [68]. Breast MRI with CAD showed high diagnostic performance for CAD diagnosis of mass lesions, but poor performance for NME, according to a 2010 report [69]. Recently, by adding radiomics signatures, discriminability with sensitivity of 0.887–0.820 and specificity of 0.80–0.864 has been confirmed even for NME [68, 70]. It is a future challenge to see how the introduction of objective and uniform criteria based on advances in image analysis technology will affect second-look indications.
